# Preparation and Crystal Structure of a Platinum(II) Complex of [CH_2_N(CH_2_COOH)CH_2_CONH_2_]_2_, the Hydrolysis Product of an Anti-Tumour Bis(3,5-Dioxopiperazin-1-YL)Alkane

**DOI:** 10.1155/MBD.1994.321

**Published:** 1994

**Authors:** Kevin B. Nolan, Leo P. Ryan, Colm J. Campbell, Patrick McArdle, Desmond Cunningham, Tim Higgins

**Affiliations:** 1 Department of Chemistry Royal College of Surgeons in Ireland St. Stephen's Green Dublin 2 Ireland; 2 Department of Chemistry University College Galway Ireland

## Abstract

The synthesis and crystal and molecular structures of the platinum(II) complex Pt(HL)Cl where H_2_L is the diacid diamide –[CH_2_N(CH_2_COOH)CH_2_CONH_2_]_2_, a hydrolytic metabolite of an antitumour active bis(3,5-dioxopiperazin-1-yl)alkane are reported. The complex is square planar and contains HL^–^  as a tridentate 2N (amino), O (carboxylate) donor. The metal to ligand bond distances are Pt-Cl 2.287(1) Å, Pt-O 2.002 (1) Å, Pt-N_trans Cl_ 2.014(1) Å and Pt-N_trans O_ 2.073 Å. There is extensive hydrogen bonding, each molecule of Pt(HL)Cl being intermolecularly hydrogen bonded to ten others giving a 3-dimensional network. There is also one intramolecular H-bond.


### PREPARATION AND CRYSTAL STRUCTURE OF A PLATINUM(II) COMPLEX OF $[CH_2N(CH_2COOH)CH_2CONH_2]_2$, THE HYDROLYSIS PRODUCT OF AN ANTI-TUMOUR BIS(3,5-DIOXOPIPERAZIN-1-YL)ALKANE

Kevin B. Nolan*[1], Leo P. Ryan[1], Colm J. Campbell[1], Patrick McArdle[2], Desmond Cunningham[2] and Tim Higgins[2]

[1] Department of Chemistry, Royal College of Surgeons in Ireland, St. Stephen's Green, Dublin 2, Ireland
[2] Department of Chemistry, University College, Galway, Ireland.

### Abstract

The synthesis and crystal and molecular structures of the platinum(II) complex Pt(HL)Cl where $H_2L$ is the diacid diamide $-[CH_2N(CH_2COOH)CH_2CONH_2]_2$, a hydrolytic metabolite of an antitumour active bis(3,5-dioxopiperazin-l-yl)alkane are reported. The complex is square planar and contains $HL^-$ as a tridentate 2N (amino), O (carboxylate) donor. The metal to ligand bond distances are Pt-Cl 2.287(1) Å, Pt-O 2.002 (1) Å, $Pt\text{-}N_{trans\ Cl}$ 2.014(1) Å and $Pt\text{-}N_{trans\ O}$ 2.073 Å. There is extensive hydrogen bonding, each molecule of Pt(HL)Cl being intermolecularly hydrogen bonded to ten others giving a 3-dimensional network. There is also one intramolecular H-bond.

### Introduction

The bis(3,5-dioxopiperazin-l-yl)alkanes (**I**) are a family of antitumour drugs of which the propane derivative is the most effective and is marketed under the name Razoxane, R = Me.[1-3] These drugs were synthesised in the expectation that they would enter cells and then undergo intracellular hydrolytic metabolism to chelating agents which would interfere with metalloenzymes essential for tumour cell growth. There are a number of possible hydrolytic metabolites one of which is the bis-acid, bis-amide, $H_2NOCCH_2(HOOCCH_2)NCH_2CH(R)N(CH_2COOH)CH_2CONH_2$, $H_2L$.

*Author to whom correspondence should be addressed.

We decided to synthesise platinum(II) complexes of ligands of this type for testing as antitumour agents and in this paper report on the preparation, properties and crystal structure of Pt(HL)Cl, where R = H.

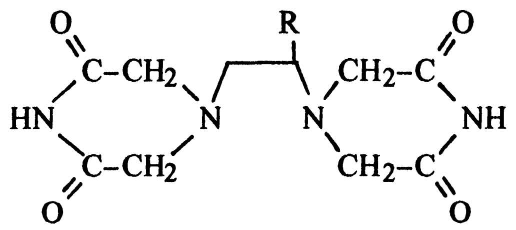

(I)

### Experimental

The ligand $H_2L$, N,N'-dicarboxamidomethyl-N,N'-dicarboxymethyl-1,2-diaminoethane, was prepared by a literature reported method for similar compounds,[4] as follows. 1.2-Bis(3,5-dioxopiperazin-l-yl)ethane (20.0 g, 79 mmol),[5] and copper(II) acetate monohydrate (15.0 g, 75 mmol) in water (20 $cm^3$) were stirred together at room temperature for 2 days to give a blue solution which was then heated under reflux for 4 hours. The solution was evaporated under reduced pressure until 10 $cm^3$ remained and this on standing at room temperature gave a blue crystalline complex. This was collected by suction filtration and dried at room temperature. The product (17.5 g, 50 mmol) which is the copper(II) complex of the title ligand, was dissolved in hot water (250 $cm^3$) and acetic acid (5.00 $cm^3$) was added to the solution which was then saturated with hydrogen sulphide. The precipitated cupric sulphide was removed by gravity filtration. The filtrate was passed through a column containing Kieselguhr and evaporated under reduced pressure to give a yellow oil. The oil was triturated and twice recrystallised from aqueous methanol-isopropanol to give $H_2L$ as a colourless solid. Yield 9.9 g (34.1 mmol), 69% from the copper(II) complex. m.p. 168$^o$ C (dec). Microanalysis: Found: C 41.15, H 6.35, N 19.24%. $C_{10}H_{18}N_4O_6$ requires C 41.38, H 6.25, N 19.30.

The complex Pt(HL)Cl was prepared as follows. $H_2L$ (0.31 g, 1.1 mmol) was dissolved in deionized water (15 $cm^3$) by heating to 60$^o$C for 5 minutes, then allowed to cool to 30$^o$C and treated dropwise, whilst stirring, with a solution of $K_2[PtCl_4]$ (0.43 g, 1 mmol) in water (5 $cm^3$). After 12 days at room temperature yellow crystals of Pt(HL)Cl were obtained, these were filtered under suction and dried at room temperature. Yield 0.33 g, 64%. Microanalysis: Found: C 22.58, H 3.35, N 10.41 Cl 6.81%. $C_{10}H_{17}N_4O_6PtCl$ requires C 23.09, H 3.30, N 10.77, Cl 6.83.

Spectroscopy

IR spectra were recorded as Nujol mulls between NaCl plates on a Philips PU 9714 spectrophotometer and UV-VIS spectra were recorded on a Philips 89730 UV/VIS scanning spectrophotometer.

X-ray Crystallography

A rectangular shaped crystal measuring 0.32 x 0.25 x 0.33 mm was selected and used for data collection, the results of which are summarised in Table 1. X-ray data were obtained on an Enraf-Nonius CAD4F diffractometer using monochromated $MoK_{\alpha}$ radiation $\lambda$= 0.7093 Å. The structure was solved by direct methods, SHELX 86,[6] and refined by full matrix least squares using SHELX 76.[7] Data were corrected for Lorentz and polarization effects and for absorption.[8] Hydrogen atoms were included in calculated positions with fixed thermal parameters on the methylene carbon atoms only. The non-hydrogen atoms were refined anisotropically. The atomic scattering factors for non-hydrogen and hydrogen atoms and the anomalous dispersion correction factors for non-hydrogen atoms were taken from the literature.[9-11] All calculations were performed on a VAX 6610 computer. The ORTEP program was used to obtain the drawings.[12]

### Results and Discussion

The addition of a solution $K_2[PtCl_4]$ to a solution of $H_2L$ produced crystals of the complex Pt(HL)Cl, the crystal and molecular structure of which shows that the geometry around the metal is approximately square planar and that HL is present as a tridentate 2N (amine), O (carboxylate) ligand. The IR spectrum of the complex in the C=O stretching region shows bands at 1730 $cm^{-1}$ (unionized COOH group), 1670 $cm^{-1}$ (amide I band) and 1630 $cm^{-1}$ (coordinated $COO^-$).[13] The UV spectrum of an aqueous solution of the complex shows bands at 350 (15.3), 24.6 (93) and 230 (344) nm with $\varepsilon$ values in $dm^3\ mol^{-1}\ cm^{-1}$ given in parenthesis.

**Table 1.** Summary of X-ray diffraction data.

| | |
|---|---|
| Formula | $C_{10}H_{17}ClN_4O_6Pt$ |
| Crystal size (mm) | 0.32 x 0.25 x 0.33 |
| M (a.m.u.) | 519.811 |
| Crystal system | Monoclinic |
| Space group | $P2_1/c$ |
| a (Å) | 6.631 (1) |
| b (Å) | 12.407 (2) |
| c (Å) | 17.470 (2) |
| β (°) | 96.43 |
| U (Å$^3$) | 1428 (1) |
| Z | 4 |
| Dc g cm$^{-3}$ | 2.417 |
| μ cm$^{-1}$ | 96.32 |
| F000 | 992 |
| Radiation | Mo $K_\alpha$ |
| Graphite Monochromator | λ = 0.7093 Å |
| Diffractometer | Enraf-Nonius CAD4F |
| Orienting Reflections, Range | 25, 13 < Θ < 20° |
| Temperature (°C) | 22 |
| Scan method | ω-2Θ |
| Data Collection Range | 2 < 2Θ < 64° |
| No. unique data | 3506 |
| Total 1 > 3 σ 1 | 3099 |
| No. of parameters fitted | 200 |
| Transmission factors, max/min | 1.28/0.86 |
| $R^a$, $R_w^b$ | 3.72%, 4.26% |
| Quality-of-fit indicator$^c$ | 2.49 |
| Largest Shift/esd, final cycle | < 0.001 |
| Largest positive peak (e/Å$^3$) | 2.06 |
| Largest negative peak (e/Å$^3$) | -1.10 |

$^aR = [\Sigma| |F_o| - |F_c| |]/\Sigma|F_o|$

$^bR_w = [[\Sigma w(|F_o - F_c|)^2]/[\Sigma w(|F_o|)^2]]^{1/2}$; $w = 1/[(\sigma F_o)^2 - 0.001019 * F_o^2]$

$^c$Quality-of-fit = $[\Sigma w(|F_o| - |F_c|)^2/(N_{obs} - N_{parameters})]^{1/2}$

In Fig. 1 is shown an ORTEP diagram of the complex with selected bond and interatomic distances and angles listed in Table 2. The Pt-Cl bond distance (2.287 Å) is similar to that found in other complexes such as cis and trans-$[Pt(NH_3)_2Cl_2]$ for which Pt-Cl = 2.33 Å and 2.32 Å respectively,[14] $K[Pt(NH_3)Cl_3]{\cdot}H_2O$ where Pt-Cl = 2.31 Å (the 3 Pt-Cl bond lengths are identical within experimental error),[15] and $[PtCl_4]^{2-}$ where Pt-Cl = 2.317 Å.[16] The Pt-O bond distance is 2.002 Å which is the same as that reported for the Pt-O bond in trans-bis(glycinato)platinum(II).[17] Since metal to ligand bond distances in platinum(II) complexes depend on the nature of the donor atom and also on the ligand trans to the leaving group as defined by the trans influence,[16] the identical values of the Pt-O bond distances quoted is not surprising since both are carboxylate oxygen atoms and both are trans to nitrogen atoms, albeit primary in one case and tertiary in the other. The C=O and C-O bond distances in the coordinated carboxylate groups are 1.237 Å and 1.287 Å respectively for Pt(HL)Cl which compares with 1.231 Å and 1.290 Å for $Pt(Gly)_2$. The Pt-N bond distances in Pt(HL)Cl are 2.014 Å for the bond trans to $Cl^-$ and 2.073 Å for the bond trans to $COO^-$. Since the nitrogen atoms are very similar, both being tertiary and both attached to three $CH_2$ groups the difference in bond distances may be partly due to the higher trans influence of $COO^-$ relative to $Cl^-$ and also to the fact that the N trans to $Cl^-$ is part of two chelate rings while the other N is involved in only one such ring.

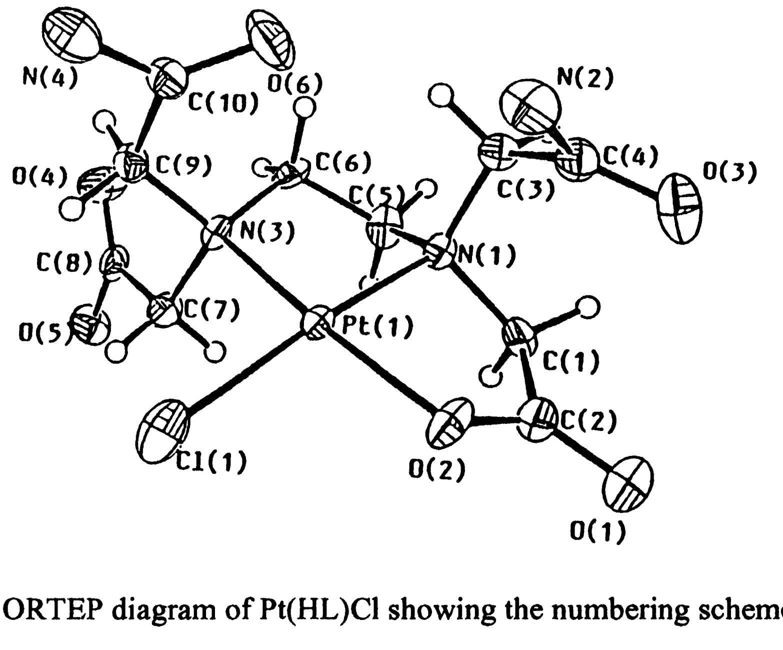

**Fig. 1** An ORTEP diagram of Pt(HL)Cl showing the numbering scheme used.

The molecular structure of Pt(HL)Cl shows that there is extensive hydrogen bonding, each molecule being involved in intermolecular hydrogen bonds to ten other molecules, giving rise to a 3-dimensional network. In Fig 2 is shown a typical molecule viewed down the x-axis and for clarity shows its contacts with only 8 other molecules. The remaining two contacted molecules lie above and below the plane. There is one intramolecular hydrogen bond formed between N(2) and O(6) which has a distance of 3.086 Å. The hydrogen bonds distances are shown in the Table 3.

**Table 2.** Selected Bond and Interatomic Distances (Å) and Angles (°) in Pt(HL)Cl.

| | | | |
|---|---|---|---|
| Pt(1) - Cl(1) | 2.287 (1) | Pt(1) - O(2) | 2.002 (4) |
| Pt(1) - N(1) | 2.014 (4) | Pt(1) - N(3) | 2.073 (4) |
| Pt(1) - C(1) | 2.784 (6) | Pt(1) - C(2) | 2.761 (6) |
| Pt(1) - C(5) | 2.778 (5) | Pt(1) - C(6) | 2.866 (5) |
| Pt(1) - C(7) | 2.891 (5) | O(1) - C(2) | 1.237 (8) |
| O(2) - C(2) | 1.287 (8) | O(3) - C(4) | 1.229 (8) |
| O(2) - C(8) | 1.184 (8) | O(5) - C(8) | 1.308 (8) |
| O(6) - C(10) | 1.217 (8) | N(1) - C(1) | 1.496 (7) |
| N(1) - C(3) | 1.490 (7) | N(1) - C(5) | 1.501 (7) |
| N(2) - C(4) | 1.332 (8) | N(3) - C(6) | 1.544 (6) |
| N(3) - C(7) | 1.522 (7) | N(3) - C(9) | 1.498 (7) |
| N(4) - C(10) | 1.347 (7) | C(1) - C(2) | 1.55 (1) |
| C(3) - C(4) | 1.510 (8) | C(5) - C(6) | 1.507 (8) |
| C(7) - C(8) | 1.536 (8) | C(9) - C(10) | 1.492 (8) |
| | O(2) - Pt(1) - Cl(1) | 93.8 (1) | |
| | N(1) - Pt(1) - O(2) | 83.6 (2) | |
| | N(3) - Pt(1) - N(1) | 88.1 (2) | |
| | N(3) - Pt(1) - Cl(1) | 94.3 (1) | |
| | N(3) - Pt(1) - O(2) | 170.5(2) | |
| | N(1) - Pt(1) - Cl(1) | 176.5 (1) | |

The fractional atomic coordinates for the non-hydrogen atoms are listed in Table 4. Additional material available from the Cambridge Crystallographic data centre includes a full listing of the remaining bond angles, H-atom coordinates and thermal parameters.

**Table 3.** Hydrogen bond distances in Pt(HL)Cl.

| Hydrogen bonds | Distance, Å |
|---|---|
| N(2)-O(3) | 2.891 |
| N(2)-O(5) | 3.080 |
| N(2)-O(6), (intra) | 3.086 |
| N(4)-O(2) | 3.032 |
| N(4)-O(4)' | 3.080 |
| O(1)-O(5) | 2.752 |

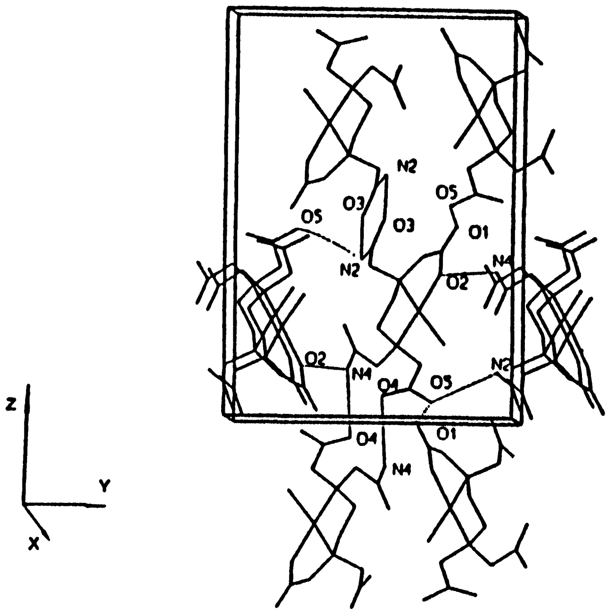

**Fig. 2.** A view along the x-axis showing intermolecular contacts.

**Table 4.** Fractional atomic coordinates x $10^4$ (Å) for Pt(HL)Cl.

| Atom | x | y | z | Atom | x | y | z |
|---|---|---|---|---|---|---|---|
| Pt(1) | 1622.7(3) | 6723.0(2) | 2654.4(1) | C(1) | -294(11) | 6941(5) | 3984(4) |
| Cl(1) | 3684 (2) | 7543(1) | 1864(1) | C(2) | 1799(11) | 7495(5) | 4143(3) |
| O(1) | 2285(10) | 7983(5) | 4753(3) | C(3) | 384(9) | 5029(4) | 3744(3) |
| O(2) | 2981(7) | 7423(3) | 3609(2) | C(4) | 2404(10) | 5054(5) | 4238(4) |
| O(3) | 2548(7) | 5442(5) | 4889(3) | C(5) | -2198(8) | 5963(5) | 2894(3) |
| O(4) | -3249(8) | 5658(4) | 469(3) | C(6) | -1810(8) | 5390(5) | 2164(3) |
| O(5) | -3763(7) | 7400(4) | 311(3) | C(7) | -1196(9) | 6933(4) | 1276(3) |
| O(6) | 2083 (8) | 4106(4) | 2295(3) | C(8) | -2838(10) | 6562(5) | 641(3) |
| N(1) | -204(6) | 6088(3) | 3384(2) | C(9) | 903(9) | 5293(5) | 1261(3) |
| N(2) | 3980(8) | 4626(4) | 3937(3) | C(10) | 2308(9) | 4485(5) | 1667(3) |
| N(3) | -217(7) | 6011(4) | 1757(2) | | | | |
| N(4) | 3846(8) | 4191(4) | 1270(3) | | | | |

### Acknowledgement

We thank the Association for International Cancer Research, the Research Committee of the Royal College of Surgeons in Ireland, EOLAS (Dublin) and the EC Human Capital and Mobility Program for supporting this research. We thank Johnson Matthey PLC for a generous loan of $K_2[PtCl_4]$.

### References

1 N. Nic Daeid, K.B. Nolan and L.P. Ryan, *J. Chem. Soc., Dalton Trans.,* 1991, 2301.
2 K.B. Nolan, L.P. Ryan and E. Bermejo-Gonzalez, *Inorg. Chim. Acta,* 1994, **215**, 55.
3 A.M. Creighton, K. Hellmann and S. Whitecross, *Nature (London),* 1969, **222**, 384.
4 Z-X Huang, P.M. May, K.M. Quinlan, D.R. Williams and A.M. Creighton, *Agents and Actions*, 1982, **12**, 536
5 A.M. Creighton, Br. Pat., 1234935, 1971.
6 G.M. Sheldrick, SHELX 86, *A Computer Program for Crystal Structure Determination*, University of Gottingen 1986.
7 G.M. Sheldrick, SHELX 76, *A Computer Program for Crystal Structure Determination*, University of Cambridge (Cambridge England) 1976.
8 N. Walker and D. Stuart, *Acta Crystallogr.,* Section A, 1983, A39, 158.
9 D.T. Cromer and J.B. Mann, *Acta Crystallogr.,* Section A, 1968, A24, 321.
10 R.F. Stewart, E.R. Davidson and W.T. Simpson, *J. Chem. Phys.,* 1965, **42,** 3175.
11 D.T. Cromer and D.J. Liberman, *J. Chem. Phys.*, 1970, **53**, 1891.
12 C.K. Johnson, ORTEP, Oak Ridge Nati. Lab. Report, ORNL (US), 1965, 3794 revised 1971.
13 K. Nakamoto and P.J. McCarthy, *'Spectroscopy and Structure of Metal Chelate Compounds'*, Wiley, New York, 1968, ch. 4.
14 G.H.W. Milburn and M.J. Truter, *J. Chem. Soc., (A)*, 1966, 1609.
15 Y.P. Jeannin and D.R. Russell, *Inorg. Chem.,* 1970, **9**, 778.
16 K.F. Purcell and J.C. Kotz, *'Inorganic Chemistry',* Saunders, Philadelphia, 1977, pp 703-706.
17 H.C. Freeman and M.L. Golomb, *Acta Crystallogr.,* Section B, 1969, B25, 1203.

**Received: March 18, 1994 - Accepted: April 19, 1994 - Received in revised camera-ready format: May 2, 1994**

